# A structured approach to promote equity in spatial accessibility to TB services during private sector engagement

**DOI:** 10.5588/ijtldopen.25.0599

**Published:** 2026-04-13

**Authors:** Y. Xiong, A.K. Millones, C.-C. Huang, E. Zavala-Abriojo, H. Campos, J. Jimenez, D.R. Jordan, L. Lecca, H.E. Jenkins, C.M. Yuen

**Affiliations:** 1 Division of Global Health Equity, Brigham and Women’s Hospital, Boston, MA, USA;; 2 Socios En Salud Sucursal Peru, Lima, Peru;; 3 Department of Global Health and Social Medicine, Harvard Medical School, Boston, MA, USA;; 4 Department of Biostatistics, Boston University School of Public Health, Boston, MA, USA.

**Keywords:** tuberculosis, Peru, health services accessibility, public–private sector partnerships, urban health services

## Abstract

**BACKGROUND:**

We sought to quantify the potential impact of private sector engagement on the equity of spatial accessibility to TB services in Lima, Peru.

**METHODS:**

We used the enhanced two-step floating catchment area (E2SFCA) method to calculate census spatial accessibility indices for access to health facilities providing clinical evaluation, sputum testing, or chest radiography in two districts. We compared different hypothetical interventions of engaging private facilities to a baseline including only public facilities. We defined an equity-promoting intervention as one that improves the median spatial accessibility index while decreasing the interquartile range (IQR).

**RESULTS:**

Our analysis included 22 public and 38 private facilities. For clinical evaluation and sputum testing, which are universally available in public facilities, neither broad nor targeted private sector engagement would have substantial impact on the IQR of the spatial accessibility index in either district. For radiography services, which are limited in public facilities, we identified equity-promoting private sector engagement interventions that would increase median spatial accessibility by 8%–28% while reducing the IQR by 1%–26%.

**CONCLUSION:**

Targeted private sector engagement can sometimes promote equity in spatial accessibility to TB services. An E2SFCA analysis provides an objective approach to identifying equity-promoting engagement interventions.

In countries with the highest TB burdens, the majority of people with TB first seek care in the private sector, and the private sector may provide treatment for up to half of all people with TB.^1^ The WHO has recommended that national TB programmes engage private sector providers to improve TB service delivery since 2001.^2^ Private sector engagement programmes in Asia and Africa have been shown to improve TB diagnosis and treatment outcomes.^3^ These programmes have involved supportive collaborations in which the national TB programme provides training, technical assistance, medications, diagnostic supplies, or equipment to private providers.^3^ However, while supporting private providers can increase overall health system capacity for delivering TB services, it can exacerbate disparities in access to TB services. One aspect of access in which these disparities can occur is spatial. If private sector services tend to be located in wealthier or more developed areas, then private sector engagement could disproportionately increase access for people who already have more convenient access to public health facilities.^4,5^ To avoid this outcome, structured approaches to assessing the spatial distribution of public and private sector services are important for promoting equity when planning private sector engagement programmes. ‘Floating catchment area’ analyses quantify spatial accessibility to health services by considering both the distance people travel to health services and the amount of the service available per unit population.^6^ They have been widely used to describe spatial accessibility to health services,^6^ which can be a first step to prioritising areas with low spatial accessibility for new health care infrastructure.^7,8^ Few examples of floating catchment area analyses exist for TB services.^5,9^

We sought to assess whether comparing changes in the median and interquartile range (IQR) of the spatial accessibility index produced by an enhanced two-step floating catchment area (E2SFCA) analysis^10^ could help identify private sector engagement strategies that promote equity in spatial accessibility to TB services.

## METHODS

Peru has an estimated TB incidence of 173 per 100,000 population,^11^ and 55% of case notifications come from the capital, Lima. This analysis focuses on two districts referred to as District A (Carabayllo, population 424,000, on the periphery of Lima) and District B (Rimac, population 183,000, adjacent to downtown Lima). In Peru, free public sector health services are delivered to 87% of the population by the Ministry of Health and EsSalud, an employer-funded insurance programme under the Ministry of Labor, which both operate their own health facilities.^12^ Private sector facilities provide services to people who pay out-of-pocket or possess private insurance; some serve EsSalud patients as well. In Lima, around 40% of people with TB first seek care in the private sector.^13,14^ However, there is no formal collaboration between the public and private sectors, and TB diagnoses made in the private sector are repeated in the public sector to become official, posing barriers to prompt treatment.^15^ Only the public sector provides TB treatment, and there is little interest from the private sector to do so.^15^

Although Latin America has limited experience with private sector engagement to deliver TB services, strategies used in Asia and Africa could be appropriate for adaptation.^3,16^ Expanding universal health coverage programmes to fully cover or subsidise private sector services, training and oversight by the national TB programme to ensure the quality of private sector TB diagnostic services, establishing formal intersectoral referral protocols, and incentivising TB diagnosis and referral from the private sector could reduce barriers to diagnosis and treatment. However, the impact of such initiatives on the equity of access to diagnostic services would depend in part on the locations of the private health facilities involved.

### Health facilities included in analysis

We included health facilities located within the areas covered by the 2017 census in District A or District B in which the public can access the key TB diagnostic services of clinical evaluation, sputum testing, and chest radiography. We included as public health facilities those operated by the Ministry of Health or EsSalud. We included as private health facilities any non-government health facility offering general medical or laboratory services, excluding those that only offered specialty services like dentistry or psychology because a person would not seek care there for TB symptoms. We included hospitals in the Sistema Metropolitano de la Solidaridad (SISOL) public–private partnership network as private facilities because patients must pay for services.^17^

We obtained lists of public health facilities from the websites of the Ministry of Health and EsSalud, confirming with the relevant authorities which facilities offered sputum testing and radiography. We identified private facilities based on a 2022 registration list from the National Health Regulatory and Supervisory Agency. We excluded from the list those whose names indicated a non-relevant medical specialty, then visited the remaining facilities to determine if they were operational and whether they offered general clinical evaluations, sputum testing, and chest radiography. We captured GPS coordinates for all health facilities.

### Spatial accessibility index

We calculated the spatial accessibility index for each census tract (generally corresponding to a city block) using the E2SFCA method.^10^ By considering both distance to health facilities and density of health facilities per population, the spatial accessibility index increases for areas closer to health facilities and those with more health facilities per population. For the distance component of the approach, we mapped the pedestrian road network travel distance from the centroid of each tract to each health facility in ArcGIS Pro v3.1.0 (ESRI, Redlands, CA, USA), applying a Gaussian decay function to weight distances of 1.00, 0.75, 0.08, and 0.00 for distances of 0–1, 1–2, 2–3, and over 3 km, respectively. For population weighting, we used census tract-level populations from the 2017 census, obtained from the National Institute for Statistics and Informatics.^18^

We compared summary measures of the spatial accessibility index for different scenarios (described below). We used the population-weighted median census tract-level spatial accessibility index for the district as a measure of overall spatial accessibility to TB services, with higher values indicating better access. We used the IQR of this index (defined as the difference between the 25th and 75th percentiles) as a measure of equality, as it quantifies the difference in accessibility between tracts with better and worse access; lower IQRs indicate greater equality. We chose this approach rather than computing the Gini coefficient, as has previously been used as a measure of inequality in floating catchment area analyses,^19,20^ because reporting the 25th, 50th, and 75th percentiles more transparently describes changes in the magnitude and distribution of spatial accessibility.

### Impact of broad and targeted private sector engagement

We first assessed whether broad engagement of all private sector facilities could be equity-promoting in terms of spatial accessibility to clinical evaluation, sputum testing, and radiography. A baseline scenario for each type of service considered only public facilities, while a broad engagement scenario added all the private facilities offering the indicated service. For each district and service type, we calculated the percentage change in the median and the IQR between the two scenarios. Any addition of services via engagement of private health facilities will increase the spatial accessibility index for at least some people. We defined an equity-promoting intervention as one that improves the district’s median spatial accessibility index while decreasing the IQR, as the only way that this can happen is for access to increase more for the bottom quartile than for the middle 50%. We then assessed whether targeted engagement of a subset of facilities would be better for promoting equity. Using the same approach described above, we assessed the impact on the median and IQR of the spatial accessibility index resulting from adding all possible subsets of private facilities to the baseline scenario of public facilities. To identify the subsets that were most equity-promoting, we identified those that decreased the IQR the most; if multiple subsets had the same impact on the IQR, we considered the one that increased the median the most. All calculations were performed using R version 4.5.1^21^.

### Ethical statement

Ethical approval was not required as no data from human subjects were used.

## RESULTS

Our analysis included 22 public and 38 private facilities across the two districts (Table 1). All public facilities offered clinical evaluation and sputum testing, and 8 (36%) offered radiography. All private facilities offered clinical evaluation, except for three private laboratories. Sputum testing was available in 21 (55%) private facilities and radiography in 8 (21%). In the baseline scenario considering only public health facilities, the median spatial accessibility index for clinical evaluation and sputum testing was 44 × 10^−6^ (IQR 39 × 10^−6^) in District A and 49 × 10^−6^ (IQR 18 × 10^−6^) in District B (Table 2). The median spatial accessibility index for radiography was lower in both districts: 10 × 10^−6^ (IQR 19 × 10^−6^) in District A and 24 × 10^−6^ (IQR 37 × 10^−6^) in District B.

**Table 1.** TB diagnostic service availability at public and private health facilities included in analysis.

Services offered	District A		District B	
	Public (N = 12)	Private (N = 22)	Public (N = 10)	Private (N = 16)
Clinical evaluation	12 (100%)	22 (100%)	10 (100%)	13 (81%)
Sputum testing	12 (100%)	13 (59%)	10 (100%)	8 (50%)
Chest radiography	4 (33%)	6 (27%)	4 (40%)	2 (13%)

**Table 2.** Impact of broad private sector engagement on population-weighted median and interquartile range of the spatial accessibility index.

District, type of service	Public		Public + Private		Change in median	Change in IQR
	Median (1st, 3rd quartile)^A^	IQR^A^	Median (1st, 3rd quartile)^A^	IQR^A^		
District A, clinical evaluation	44 (17, 56)	39	117 (66, 163)	97	+166%	+150%
District A, sputum test	44 (17, 56)	39	85 (53, 116)	63	+94%	+62%
District A, chest radiography	10 (1, 20)	19	36 (6, 53)	47	+260%	+145%
District B, clinical evaluation	59 (52, 69)	18	140 (102, 168)	66	+138%	+274%
District B, sputum test	59 (52, 69)	18	106 (89, 128)	39	+70%	+121%
District B, chest radiography	24 (3, 40)	37	40 (16, 51)	35	+69%	−5%

IQR = interquartile range.

A All values of the spatial accessibility index have been multiplied by 10^6^.

In both districts, broad engagement of private sector facilities would increase the median spatial accessibility indices for clinical, sputum testing, and radiography by 68%–260%. However, only one broad engagement scenario met our definition of an equity-promoting intervention: in District B, broad engagement of the two private facilities with radiography services would increase the median spatial accessibility index by 68% while reducing the IQR by 5%. In all other cases, broad engagement of private facilities would increase the IQR by 62%–274%, suggesting increased disparity in spatial accessibility.

In District A, targeted engagement of seven of the private facilities offering clinical evaluation and five offering sputum testing would meet our definition of equity-promoting interventions, increasing the median spatial accessibility by 51% and 38%, respectively, while reducing the IQR by <1% (Table 3). Engaging one private facility offering radiography would increase the median spatial accessibility by 8% while reducing the IQR by 1%. In District B, no private facilities (either alone or in combination) would promote equity in spatial accessibility to clinical evaluation or sputum testing. Engaging the two private facilities that offer radiography services alone or in combination would be equity-promoting interventions, as both are located in areas whose public facilities lack radiography services. The largest reduction in IQR (−26%) would come from engaging a single private facility, although the increase in median accessibility (+28%) would be lower than what would be achieved by engaging both (Tables 2 and 3).

**Table 3.** Impact of targeted engagement of the subset of private facilities that would achieve the largest decrease in the population-weighted interquartile range of the spatial accessibility index.

District, type of service	Number of private facilities in subset	Median (1st, 3rd quartile)^A^	IQR^A^	Change in median	Change in IQR
District A, clinical evaluation	7	66 (45, 84)	39	+51%	−<1%
District A, sputum test	5	61 (36, 75)	39	+38%	−<1%
District A, chest radiography	1	11 (2, 22)	19	+8%	−1%
District B, clinical evaluation	No equity-promoting subset	Not applicable			
District B, sputum test	No equity-promoting subset	Not applicable			
District B, chest radiography	1	30 (14, 41)	27	+28%	−26%

IQR = interquartile range.

A All values of the spatial accessibility index have been multiplied by 10^6^.

The Figure illustrates how broad engagement of all private facilities in District A would exacerbate disparities in access to clinical evaluation by creating a few zones of much higher spatial accessibility that are mostly in areas well served by the public sector. Targeted engagement would increase spatial accessibility for some areas that are poorly served by the public sector and avoid creating high-access zones. However, there are several parts of the district with neither public nor private facilities.

**Figure.**
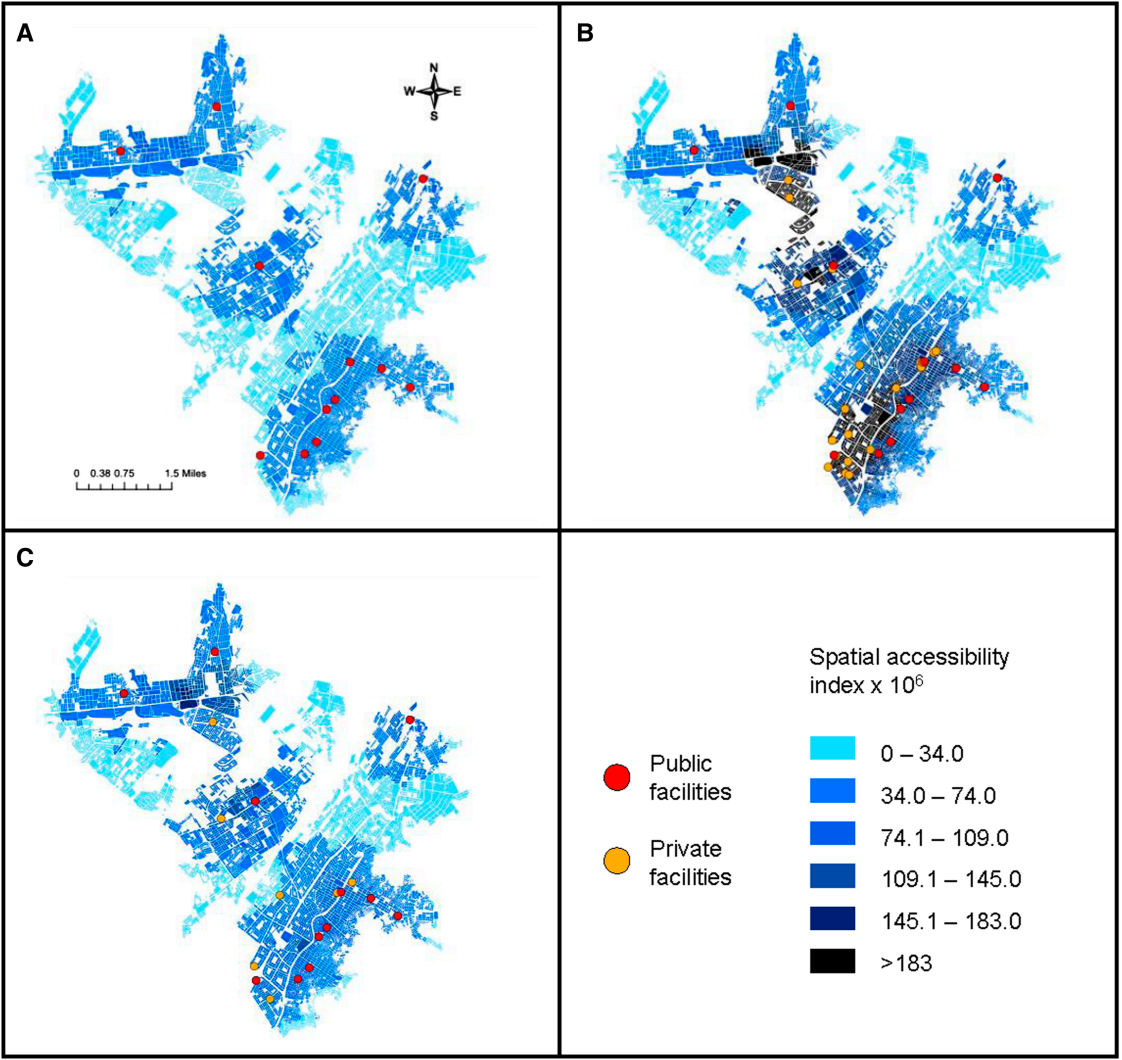
Census tract-level spatial accessibility indices reflecting access to TB clinical evaluation in District A considering (**A**) only public health facilities, (**B**) broad engagement of private facilities, and (**C**) targeted engagement of seven private facilities.

## DISCUSSION

In Lima, Peru, E2SFCA analyses in two districts suggested that broadly engaging private facilities would increase disparities in access to TB services. For clinical evaluation and sputum testing, which are universally available in the public sector, we were unable to identify private facilities whose engagement would substantially reduce disparities in spatial accessibility. There were some scenarios in which partnering with private facilities could help to reduce disparities in spatial accessibility to chest radiography services, which are limited in the public sector.

While private sector engagement initiatives have improved TB case detection, their impact on equity of access to care is not routinely assessed. Even in settings where large proportions of people with TB seek care first in the private sector, the individuals who do so may tend to be wealthier than those who do not.^22,23^ This may occur because wealthier individuals are better able to pay required service fees in the private sector^24^ or because private facilities catering to a paying clientele are located where wealthier people live.^25^ In our study, most private facilities were located in areas that were already well served by the public sector. Thus, interventions such as certifying private laboratories and establishing contracts to subsidise TB diagnostic services in private facilities for patients with government insurance could increase disparities in access. Alternative solutions to improve access to TB diagnostic services in underserved communities of Lima could include mobile TB screening units,^26^ incorporating TB screening into existing outreach programmes such as immunisation campaigns,^27^ and establishing community sputum collection points in locations such as pharmacies.^28^ The analytic approach we articulate could be used to identify equity-promoting service delivery locations for these strategies.

Considering spatial accessibility to existing services is an important aspect of planning private sector engagement interventions, as distance to health services is a common barrier to prompt diagnosis.^29^ Ensuring the affordability of private sector services is also critical. Despite the for-profit nature of most private sector health facilities, it is possible to deliver affordable care in the context of public–private partnerships that regulate user fees. In Myanmar, a public–private partnership with a social franchising model established a network of private clinics providing government-subsidised TB services with user fees that were low enough to be affordable but sufficient for profit.^30^ The network was shown to disproportionately serve poorer individuals with TB in urban areas.^31^ In Lima, the public–private partnership SISOL network delivers hospital and multi-specialty services with regulated user fees.^17^ This model could potentially be leveraged to introduce TB diagnostic services into underserved areas of Lima.

Our study has limitations. There is no single measure of inequality that captures all the nuances of the distribution of a value. Minimising the IQR while increasing the median prioritises improvement in the bottom quartile relative to the middle 50% but is not sensitive to changes for the top quartile. Other measures of inequality such as a Gini coefficient^19,20^ or Thiel index^8^ could conceivably be used in place of the IQR, which would be more sensitive to different changes in the distribution of accessibility. In addition, we used pedestrian road network distance as a measure of spatial access, but distance does not capture other relevant aspects of spatial access like public transport availability, for which systematic data were not available. We used 2017 census data, and population growth since the census has not been evenly distributed geographically, creating a source of inaccuracy in our spatial accessibility estimates. This is likely to affect our results more for District A than District B, as the former but not the latter has experienced substantial population growth. Finally, the approach we propose is likely applicable to urban settings in middle-income countries (which is relevant to a substantial proportion of people with TB globally) but not necessarily to rural settings or low-income countries where private sector TB services are not widely available.

## CONCLUSION

It is possible for private sector engagement initiatives to promote equity in spatial accessibility to TB services, but this depends on the geographic distribution of private versus public sector services. Assessing changes in the distribution of the spatial accessibility index produced by an E2SFCA analysis as an equity indicator provides a method for anticipating the impact of private sector engagement on equity of spatial accessibility. This approach can help private sector engagement programmes avoid exacerbating existing disparities in access to TB services.
